# Do socio-cultural factors influence college students’ self-rated health status and health-promoting lifestyles? A cross-sectional multicenter study in Dalian, China

**DOI:** 10.1186/s12889-017-4411-8

**Published:** 2017-05-19

**Authors:** Sainyugu Lolokote, Tesfaldet Habtemariam Hidru, Xiaofeng Li

**Affiliations:** School of Public Health, Department of Epidemiology and Biostatistics, Dalian Medical University, Dalian, China

## Abstract

**Background:**

An unhealthy lifestyle of college students is an important public health concern, but few studies have been undertaken to examine the role of socio-cultural differences.

**Methods:**

For this cross-sectional comparative study, data on college students’ health-promoting lifestyles (HPL), as measured using the Health-Promoting Lifestyle Profile (HPLP-II) scale, and self-rated health status (SRH) as measured by Sub-Optimal Health Measurement Scale (SHMS V1.0) were collected from 829 college students.

**Results:**

The sample of 829 college students included 504 (60.8%) Chinese and 325 (39.2%) international students. Chinese students had higher scores in overall health-promoting lifestyle (HPL) (*P* < 0.001, eta squared =0.113) and in all the six subclasses than their international counterparts. In relation to health status evaluation, the two groups varied in physiological health (*P* < 0.001, eta squared = 0.095) and social health (*P* = 0.020, eta squared = 0.007) but there was no significant difference in psychological health subscale (*P* = 0.156, eta squared = 0.002). HPL was predicted by financial status among the Chinese group and by student’s major, age and level of education in the international group. Body mass index (BMI) and financial status emerged as predictors of the three subscales of SHMS V1.0 in the Chinese group and also of physiological and psychological subscales in the international group. Gender was associated with psychological health in both groups. Smoking status was a predictor of psychological health in both groups and also of social health in the international group. The level of education emerged as a predictor of social health in the international group.

Regression analyses revealed a significant association between health status and healthy lifestyle (*P* < 0.001). In reference to participants with “excellent” lifestyle, participants with moderate lifestyle were at a 4.5 times higher risk of developing suboptimal health status (SHS) (OR: 4.5,95% CI:2.2-9.99) and those with a ‘general’ lifestyle were at a 3.2 times higher risk SHS (OR: 3.2, 95% CI: 1.5-7.18). Good and moderate HPLP-II levels of nutrition are associated with low risk of suboptimal health status (OR: 0,41 and 0,25, respectively). Participants in good and moderate HPLP-II levels of interpersonal relations are associated with higher risk of suboptimal health (OR:2,7 and 3,01 respectively) than those in excellent levels of HPLP-II.

**Conclusion:**

Collectively, these findings provide a convincing body of evidence to support the role of socio-cultural factors as key determinants of the HPL and SRH of college students.

## Background

A health-promoting lifestyle (HPL) encompasses a multidimensional pattern of self-initiated perceptions and activities aimed to maintain and improve individual’s health and wellness [1].HPL can decrease the occurrence of disease, lower the death rate and contribute to an improved health status [2, 3]. On the contrary, risky health behaviors are actions that increase the risk of injury and disease in general [4], for instance, tobacco and alcohol use, an unhealthy diet and lack of exercise lead to various chronic diseases. Furthermore, unhealthy behavior and lifestyle are two important factors associated with ten major causes of death [5–10].

Though healthy lifestyle behaviors are important for individuals in all periods of life, this is of primary importance during youth. The period of emerging adulthood is an important age for the formation of health behaviors associated with an increased risk of chronic disease [11]. Unhealthy practices and behaviors established during young ages may resonate across a lifespan and result in increased health risks later in life [12–15]. As such, this unique developmental period may be an ideal time for the effective provision of preventive health information. Therefore, assessment of health promoting behaviors among university students is important, as they are young individuals.

Globalization has led to increased human mobility hence many students travel out of their countries to study abroad. It is estimated that over 300,000 foreign students come to mainland China on an annual basis to study in higher education institutes [16]. This figure is estimated to increase to over half-a-million international students annually by the year 2020 [16]. Non-native students are likely to face challenges as they adjust themselves to a different environment, culture, values, and attitudes, which might influence their personal health, lifestyles, and behaviors. At the same time, these individuals are exposed to a new level of freedom related to lifestyle choices (e.g., eating habits, sleep routines, levels of participation in physical activity, and alcohol/tobacco use). It is well documented that different ethnicities may place different values on health and healthy behaviors [17] and international students may confront different health challenges from those of Chinese locals due to a difference in cultural and social environments and health care systems. Research has revealed that international students generally experience more difficulties adapting to college life abroad than they would in their home countries [18]. This could be attributed to challenges due to homesickness, unfamiliar diets, language barriers, health and financial issues, relationships with peers, daily living activities, religion, and differences in the education systems.

Students native to China are also challenged by unhealthy lifestyles [19]. Over 46–56% of Chinese college students were reported to be in suboptimal health status (SHS) [7]. SHS is defined as medically undiagnosed or functional somatic syndromes [20–22]. SHS is associated with unhealthy lifestyles [7]. Individuals in SHS deteriorate in vitality, physiological function, and the capacity to adapt to varying conditions.

Previous college studies have paid little attention to the influence of culture and modern society on health-promoting lifestyles, especially in China. To the best of our knowledge, no empirical studies have examined the similarities and differences in SRH and health-promoting lifestyle in China. Such a study would fill an important gap in the literature. Hence, we carried out an investigation among college students in Dalian, China to assess SRH and HPL in an international and local sample of college students affiliated with different cultural, social and economic backgrounds. Also, the present study examined the similarities and differences in HPL and SRH between international and Chinese, and associations of health-promoting lifestyle and SRH.

The following research questions were addressed in the study:

1. Are there differences in health-promoting behaviors and self-rated health between Chinese and international college students?
2. Is there any association among health behaviors and self-rated health status?

## Methods

### Design & Participants

**Fig. 1:**
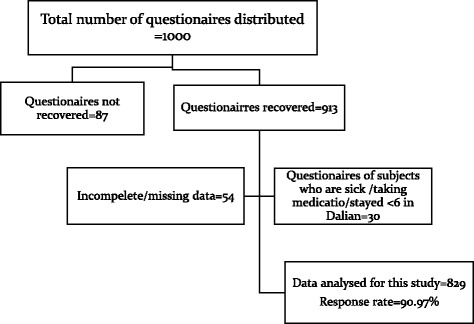
Flowchart showing inclusion and exclusion criteria of the study participants

A cross-sectional study was carried out between May and July 2016, using a self-administered questionnaire while interviewing a population of college students. The study was conducted at four conveniently selected universities in the city of Dalian. To increase participation and completeness/truthfulness of each questionnaire, recruitment was conducted during lectures. Selected participants were college students who met the following inclusion criteria: enrolled in an academic degree program at all levels, have stayed in China for longer than six months, and had no critical illness or intake of medication in the previous 3 weeks. (Fig. 1).

### Sample size

We used the formulae for two independent samples, a dichotomous outcome to estimate our sample size [23].

$$ ni=2\left(\frac{Z1-\alpha /2+ Z2\hbox{--} \beta /2}{ES}\right)\hat{\mkern6mu} 2 $$

Where ni is the sample required in each group (*i* = 1,2) α is the selected level of significance, Z1- α/2, is the value from the standard normal distribution holding 1- α/2, 1-β/2 is the selected power, and Z_2−_β/2 is the value from standard normal distribution holding 1-β below it.ES is the effect size defined as

$$ \mathrm{ES}=\frac{\mid \mathrm{P}1-\mathrm{P}2\mid }{\surd P\left(1- P\right)} $$

Where |p1-p2| is the absolute value of the difference in proportions between the two groups expected under the alternative hypothesis, H_1_ and *P* is the overall proportion, based on pooling the data from the two comparison groups. We estimated that 45 and 56% of the international and Chinese students will be in suboptimal health status, respectively. The minimum sample size required to ensure that the power of the test is 80% to detect the difference in proportions taking into account of a drop out of 30% was calculated as follows;

$$ \mathrm{ES}=\frac{\mid 0.56-0.45\mid }{\surd 0.51\Big(1-0.51}=0.22 $$

$$ ni=2\left(\frac{1.96+0.84}{22}\right)\hat{\mkern6mu} 2=324.0 $$

Sample sizes n_1_ = 324 and, n2 = 324 was needed to ensure that the test of hypothesis had 80% power to detect suboptimal health status in college students. With an estimated 30% drop data; therefore, our target population was; 648*100/70 = 926.

### Measures

#### Socio-demographic characteristics

We employed a self-administered questionnaire to obtain data on respondents’ socio-demographic information including age, gender, level of education, student’s major, smoking status, alcohol consumption, financial status, length of stay in Dalian, body weight and height which were transformed into body mass index (BMI) (kg/m^2^). This is a statistical measure of the weight of a person scaled according to their height. The following four categories of BMI included being underweight (≤18.5); normal weight (18.6–24.9); overweight (25–29.9); and obese (≥30).

### Self-rated health status (SRH)

We adopted the Sub-Optimal Health Measurement Scale Version 1.0 (SHMS V1.0) to evaluate the SRH among the college students. SHMS V1.0 has been shown to be a valid and reliable tool in various ethnic groups, with a Cronbach’s alpha and split-half reliability coefficients of 0.917 and 0.831, respectively. SHMS V1.0 comprises 39 items with 3 dimensions: physiological health (14 items), psychological health (12 items), societal health (9 items), and 4 other items for a health status evaluation, in which participants were asked: "What is your general feeling in terms of physical/psychological/social/general health?" The 35 items of five-point Likert-type (1 = never, 2 = occasionally, 3 = sometimes, 4 = often, 5 = routinely) were used to measure the respondent’s self-reported health problems. Transformed scores were determined to account for reverse questions. The total scores for each SHMS V1.0 domains were transformed to a range of 0 to 100, with the highest scores representing better SRH. The threshold values for SHS in the physiological, psychological and social health dimensions of SHMS V1.0 were fixed at 68,67, and 67 respectively. Because few subjects reported their health to be in the disease state, they were combined with the sub-optimal status group. A dichotomous variable was created (0 = Healthy, 1 = Sub-Optimal health) for self-rated health. The overall scale Cronbach’s alpha in this study was 0.873 and 0.898 for the international and Chinese students, respectively. The sub-scale alpha of SRH physiological health was 0.701 and 0.798, psychological health, 0.788 and 0.846, and societal health 0.854 and 0.798 for the international and the Chinese students respectively.

### Health-promoting lifestyle (HPL)

To evaluate the students’ lifestyle, we used the 52-item Health Promoting Lifestyle Profile II (HPLP-II). The measurement has been used widely internationally and has good validity and reliability [24]. We used a previously translated and validated Chinese version for the Chinese and an original English version for international students. It consists of 6 dimensions namely: health responsibility (9 items), spiritual growth (9 items), physical activity (8 items), nutrition (9 items), interpersonal relationships (9 items), and stress management (8 items). To determine the frequency of each behavior, a 4-point Likert scale (1 = “never”, 2 = “sometimes”, 3 = “often”, and 4 = “routinely”) was used. In line with the previous studies, a mean ≥ 2.50 was considered to be a positive response [25] indicating that one is engaging in health-promoting lifestyle. Since all the items of HPLP-II were presented positively, there were no items to be reversed. Scores for each dimension were generated by summing the scores of all items and computing a mean score, which has a possible range from 1 to 4. Following the recommendations of the original authors of the scale, the overall HPLP-II score was obtained by calculating the mean of the responses to all 52 items [1].HPLP-II scores ranged between 52 and 208. To determine the levels of HPLP-II subscales, the scores were divided by quartiles into low (1.00–2.38), moderate (2.39–2.61), good (2.62-2.91) and excellent (2.92–4.00) HPL. High scores indicated a greater frequency of health-promoting behaviors. A Cronbach’s alpha of 0,887 and 0,939 for international students and Chinese group, respectively, regarded as a reliability measure, was obtained in this study.

### Data analysis

Data were analyzed with descriptive statistics, multiple linear and binary logistics regression analysis using the Statistical Package for the Social Sciences for Windows (Version 21; SPSS Inc., Chicago, IL). Data were expressed as the mean ± SD for continuous variables or the n (%) for categorical variables. An independent *t*-test and Chi-square for independence were used to compare mean differences and proportions between the groups, respectively. Pearson/spearman rank correlation was used to explore the relationship between HPLP-II scores and SRH status. One-way analysis of variance (ANOVA) or *t*-test was performed to examine the effects of demographic characteristics on HPLP-II and SHMS V1.0 scores. Effect sizes (ESs) for independent *t*-tests and one-way ANOVA were calculated by Eta squared and Cohen’s d (0.1: small effect; 0.6: medium effect; 0.14: large) to assess the magnitude of the significant group differences [26]. Binary logistics was conducted to explore the association between SRH status and HPL among the college students. A *p*-value of ≤*0.05* was considered statistically significant.

## Results

### Characteristics of the participants

Table 1 depicts the characteristics of the 829 participants; the mean age was 22.18 (range, 18 years). Out of the total participants, 60.80% were female, and 39.20% were international students, while 60.08 were Chinese. There was a significant difference in terms of age (*n* = 829, *t* = 6.99, *P* ≤ 0.050), gender (*n* = 829, x^2^ = 14, *P* < 0.001), BMI (*n* = 829, x^2^ = 21.53, *P* < 0.001), level of education (*n* = 829, x^2^ = 23.04, *P* < 0.001), smoking status (*n* = 829, x^2^ = 58.172, *P* < 0.001), alcohol consumption (*n* = 829, x^2 =^40.862, *P* < 0.001), students’ major (*n* = 829, x^2^ = 47.117, *P* < 0.001) and financial status (*n* = 829, x^2^ = 52.23, *P* < 0.001 ).

**Table 1:** Socio-demographic characteristics of the participants

Parameters	Total sample (*n* = 829)	International students	Chinese students	x^2^/t/z
Age	23.04 ± 3.30	22.95 ± 3.90	21.63 ± 2.77	*t* = −6.49*
Gender				
Female	498(60.1%)	221 (68%)	277(55%)	×^2^ = 14.00*
Male	331 (39.9%)	104 (32%)	227(45%)	
BMI				
< 18.5	74 (8.9%)	32 (9.8%)	42 (8.3%)	×^2^ = 21.03*
18.6–24.9	596 (71.9%)	256 (78.8%)	340 (67.5%)	
25.0–29.9	119 (14.4%)	27 (8.3%)	92 (18.3%)	
≥ 30	40 (4.8%)	10(3.1%)	30 (6.0%)	
Level of Education				
Sophomore/freshman year	628 (77.0%)	245(75.4%)	393 (78.0%)	×^2^ = 23.04*
Senior year	168(20.25%)	60(18.5%)	108(21.4%)	
Post-graduate	23(2.8%)	20 (6.2%)	3(0.6)	
Students’ major				
Medical	653(78.8%)	298(91.7%	355(70.4%)	×^2^ = 53.38*
Non-Medical	176(21.2%)	27(8.3%)	149(29.6%)	
Smoking Status				
Current Smoker	73 (8.8%)	59 (18.2%)	14 (2.8%)	×^2^ = 58.172*
Non-smoker	756 (91.2%)	266 (81.8%)	490 (97.2%)	
Alcohol Consumption				
drinker	254 (30.6%)	141(43.60%)	113(22.4%)	×^2^ = 40.862*
Non-drinker	575 (69.4%)	184 (56.40%)	391 (77.6%)	
Financial Status (RMB)				
≤ 1000	221(26.7%)	104 (32.0%)	117(23.3%)	×^2^ = 52.23*
1000 ~ 2000	464(56.0%)	203 (62.5%)	261(51.9%)	
> 2000	143 (17.3%)	18 (5.5%)	126 (24.9%)	
Length of stay in Dalian (yrs.)		1(1)	2.0 (1.00)	z = −1.202*

*P﻿ ≤ 0.05

### Health-promoting lifestyle (HPL) of college students in Dalian City, China

**Fig. 2:**
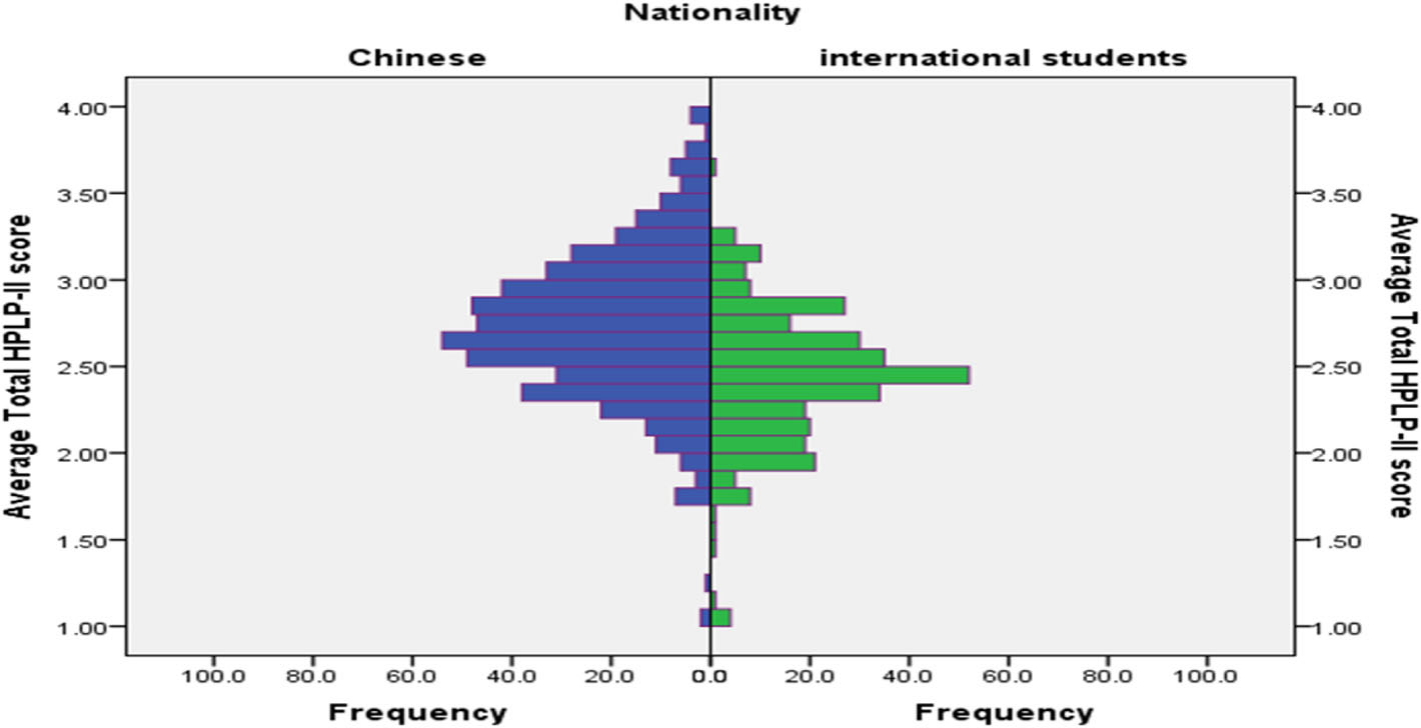
A graphical comparison on HLPLP-scores between the Chinese and international students in Dalian City, China

Average total HPLP-II scores for the Chinese and international group are shown in Fig. 2. For international students, the overall mean score was 2.44 ± 0.42, and the subscale mean scores ranged from 2.16 ± 0.43 to 2.75 ± 0.55. For Chinese students, the overall mean score was 2.74 ± 0.44, and subscale means scores ranged from 2.30 ± 0.61 to 3.00 ± 0.52. The Chinese students had significantly higher scores than the international on the average total HPLP-II (*P* < 0.001) and for each of the subscales (*P* < 0.050) (Table 2).

**Table 2:** Effects of socio-demographic characteristics on the health-promoting lifestyle (HPL) in Dalian City, China

Socio-demographic characteristics	Total HPLP-II Mean ± SD	HR Mean ± SD	SG Mean ± SD	PA Mean ± SD	Nutrition Mean ± SD	IR Mean ± SD	SM Mean ± SD
Gender							
Female	2.63 ± 0.47	2.28 ± .59	2.24 ± .60	2.33 ± 0.58	2.68 ± 0.60	2.89 ± 0.58	2.38 ± 0.50
Male	2.65 ± 0.40	2.24 ± 0.57	2.88 ± 0.54	2.34 ± 0.57	2.74 ± 0.55	2.89 ± 0.49	2.40 ± 0.47
t	−.487	1.12	−.395	−1.408	−1.11	−.079	−.398
*P* value	0.616	0.264	0.267	0.694	0.160	0.937	0.688
95% CI	-0.08 ~ .05	−0.04 ~ .13	−0.13 ~ 0.03	−0.10 ~ 0.07	−0.14 ~ 0.02	−0.08 ~ 0.07	−0.08 ~ 0.05
Eta squared	0.000	0.002	0.001	0.001	0.002	0.000	0.000
BMI							
< 18.5	2.60 ± 0.51	2.62 ± 0.63	2.85 ± 0.65	2.34 ± 0.51	2.62 ± 0.63	2.85 ± 0.69	2.41 ± 0.56
18.6 - 24.9	2.64 ± 0.44	2.70 ± 0.59	2.92 ± 0.57	2.32 ± 0.58	2.70 ± 0.59	2.90 ± 0.53	2.39 ± 0.48
25.0 - 29.9	2.64 ± 0.39	2.79 ± 0.53	2.85 ± 0.55	2.35 ± 0.57	2.79 ± 0.54	2.86 ± 0.52	2.39 ± 0.46
≥ 30	2.69 ± 0.54	2.74 ± 0.64	2.91 ± 0.63	2.44 ± 0.69	2.74 ± 0.63	2.86 ± 0.65	2.37 ± 0.64
F	0.288	1.042	0.529	0.614	1.410	0.264	0.040
*P* Value	0.834	0.373	0.662	0.606	0.239	0.852	0.989
Eta squared	0.001	0.004	0.002	0.002	0.005	0.000	0.000
Level of education							
Sophomore/Freshman year	2.63 ± 0.44	2.22 ± 0.57	2.90 ± 0.57	2.32 ± 0.58	2.70 ± 0.59	2.89 ± 0.55	2.38 ± 0.49
Senior Year	2.70 ± .45	2.43 ± .58	2.90 ± .61	2.40 ± .55	2.76 ± .58	2.91 ± .54	2.44 ± .48
Post graduate	2.47 ± 37	2.35 ± .51	2.82 ± .50	2.22 ± .57	2.40 ± 0.39	2.59 ± 0.46	2.14 ± 0.29
F	3.061	8.401	0.166	1.523	3.125	2.934	0.040
*P* value	0.047	0.000	0.847	0.219	0.044	0.054	0.989
Eta squared	0.008	0.021	0.000	0.004	0.008	0.007	0.000
Students’ major							
Medical	2.60 ± 0.43	2.25 ± 0.55	2.87 ± 0.56	2.30 ± 0.55	2.67 ± 0.58	2.85 ± 0.53	2.36 ± .49
Non-medical	2.75 ± 0.49	2.33 ± .66	2.99 ± .62	2.44 ± .65	2.85 ± .60	3.03 ± 0.58	2.50 ± 0.48
t	−3.895	−1.62	−2.62	−3.65	−2.46	−3.95	−3.68
*P* value	0.000	0.100	0.014	0.009	0.000	0.000	0.000
95% CI	−.22 ~ −0.07	−0.19 ~ 0.02	−0.22 ~ −0.02	−0.25 ~ −0.03	−0.28 ~ −0.09	−0.27 ~ −0.09	−0.23 ~ −0.07
Eta squared	0.019	0.003	0.003	0.003	0.005	0.019	0.017
Smoking status							
Current smoker	2.48 ± 0.42	2.30 ± 0.54	2.68 ± 0.52	2.33 ± 0.54	2.46 ± 0.50	2.65 ± 0.54	2.22 ± 0.47
Non-smoker	2.65 ± 0.44	2.26 ± 0.58	2.92 ± 0.58	2.33 ± 0.58	2.73 ± 0.59	2.91 ± 0.54	2.41 ± 0.49
t	−3.161	0.444	−3.412	0.013	−3.797	−3.822	−3.161
*P* value	0.002	0.657	0.001	0.990	0.000	0.000	0.002
95% CI	-0.28 ~ −0.07	−0.11 ~ 0.17	−0.38 ~ −0.10	−0.14 ~ 0.14	−0.41 ~ −0.13	−0.39 ~ −0.12	−0.28 ~ −0.07
Eta squared	0.012	0.000	0.014	0.000	0.018	0.005	0.012
Alcohol consumption							
drinker	2.56 ± 0.42	2.19 ± 0.56	2.80 ± 0.57	2.35 ± 0.55	2.58 ± 0.53	2.79 ± 0.52	2.33 ± 0.46
Non-drinker	2.67 ± 0.45	2.30 ± 0.59	2.94 ± 0.57	2.32 ± 0.59	2.76 ± 0.60	2.93 ± 0.55	2.42 ± 0.50
t	−3.335	−2.312	−3.242	0.642	−3.824	−3.357	−2.185
*P* value	0.001	0.021	0.001	0.521	0.000	0.001	0.029
95% CI	-0.18 ~ −0.05	−0.19 ~ −0.02	−0.23 ~ −0.06	−0.06 ~ 0.12	−0.26 ~ −0.08	−0.22 ~ −0.06	−0.16 ~ −0.01
Eta squared	0.014	0.007	0.013	0.000	0.018	0.014	0.006
Financial status							
≤ 1000	2.63 ± .49	2.24 ± .58	2.91 ± .66	2.34 ± .61	2.66 ± .63	2.92 ± .58	2.39 ± .54
1000 ~ 2000	2.65 ± .43	2.29 ± .59	2.91 ± .54	2.34 ± .57	2.72 ± .57	2.88 ± .54	2.40 ± .45
> 2000	2.62 ± .43	2.23 ± .55	2.85 ± .55	2.30 ± .57	2.75 ± .58	2.86 ± .53	2.35 ± .50
F	0.229	0.762	0.549	0.212	0.983	0.657	0.499
*P* value	0.795	0.467	0.578	0.809	0.375	0.519	0.607
Eta squared	0.000	0.002	0.001	0.000	0.000	0.002	0.001
Nationality							
Chinese	2.74 ± 0.44	2.30 ± 0.61	2.98 ± 0.57	2.38 ± 0.60	2.92 ± 0.54	3.00 ± 0.52	2.53 ± 0.47
International	2.44 ± 0.42	2.21 ± 0.52	2.75 ± 0.55	2.24 ± 0.52	2.34 ± 0.47	2.68 ± 0.52	2.16 ± 0.43
*P* value	0.000	0.030	0.000	0.000	0.000	0.000	0.000
95% CI	0.24 ~ 0.36	0.01 ~ 0.17	0.15 ~ 0.31	0.06 ~ 0.23	0.51 ~ 0.66	0.15 ~ .25	0.30 ~ 0.43
Eta squared	0.113	0.006	0.037	0.015	0.257	.083	0.136

### Socio-demographic characteristics and HPL of college students in Dalian City China

Overall, the average total HPLP-II score was substantially related to the level of education, smoking status, and type of institution. Healthy behaviors were observed among the senior year students (mean = 2.70, *P* = 0.050), non-smokers (mean = 2.65, *P* = 0.020), non-drinkers (mean = 2.67, *P* = 0.010) and in non-medical students (mean = 2.75, *P* < 0.001). According to HPLP-II subscales with health responsibility as the dependent variable, the highest scores were observed among senior year students (mean = 2.43*, P* < 0.001) and non-drinkers (mean = 2.30, *P* = 0.020). With spiritual growth as the dependent variable, the highest scores were observed among the non-medical students (mean = 2.99, *P* = 0.014), non-smokers *(*mean = 2.92, *P* = 0.00) and non-drinkers (mean = 2.94, *P* = 0.010). In physical activity subscale, non-medical students reported significantly higher scores (mean = 2.44, *P* = 0.009) than their medical counterparts. With nutrition subscale as the dependent variable, the highest scores were observed among non-medical students (mean = 2.85, *P* = 0.00,) non-drinkers (mean = 2.76*, P* < 0.001) and non-smokers (mean = 2.73, *P* = 0.00). In relation to interpersonal relations subscale: the highest scores were observed among non-medical students (mean = 3.03, *P* < 0.001), non-smokers (mean = 2.91 ± .54, *P* = 0.00) and non-drinkers (mean = 2.94, *P* = 0.00). In stress management subscale, better stress management strategies were observed among the non-medical (mean = 2.50, *P* < 0.001) and non-smokers students (mean = 2.41, *P* < 0.001) (Table 2).

### Comparison of self-rated health of between the Chinese and international students

The Chinese students had significantly higher scores than the international in societal health subscale (*P* < 0.001) whereas the international students had significantly higher scores than their Chinese counterparts in the physical health subscale (*P* < 0.001). There was no significant difference in the mean score of psychological health subscale between the two groups (*P* = 0.217) (Table 3).

**Table 3:** Self-rated health (SRH) status of college students in Dalian City, China

Socio-demographic characteristics	Physical health ± SD	Psychological health ± SD	Social health ± SD
Gender			
Female	68.30 ± 9.89	66.51 ± 13.53	65.52 ± 15.21
Male	66.48 ± 10.02	64.78 ± 12.40	64.70 ± 13.91
t	2.545	1.84	0.776
*P* value	0.011	0.067	0.438
95% CI	0.42 ~ 3.24	−0.12 ~ 3.58	−1.27 ~ 2.90
Eta squared	0.008	0.004	0.000
BMI			
< 18.5	69.19 ± 9.70	68.46 ± 11.87	67.18 ± 15.38
18.6 - 24.9	68.29 ± 9.99	66.65 ± 13.17	65.63 ± 14.60
25.0 - 29.9	63.27 ± 9.28	61.22 ± 11.97	62.81 ± 14.23
≥ 30	66.64 ± 9.14	62.28 ± 14.28	61.99 ± 15.45
F	9.201	7.585	2.232
*P* Value	0.000	0.000	0.083
Eta squared	0.033	0.03	0.000
Level of education			
Freshman year/sophomore	67.36 ± 9.93	65.64 ± 13.36	64.31 ± 14.96
Senior year	67.32 ± 9.65	66.15 ± 12.31	68.07 ± 12.57
Post-graduate	76.79 ± 10.61	68.98 ± 11.30	69.60 ± 14.96
F	8.008	0.637	5.104
*P* value	0.000	0.529	0.006
Eta squared (h2)	0.012	0.000	0.013
Students’ major			
Medical	67.59 ± 10.20	65.35 ± 13.06	64.41 ± 14.75
Non-medical	67.49 ± 9.16	67.46 ± 13.17	67.97 ± 14.19
t	0.112	0.709	−2.85
*P* value	0.911	0.100	0.005
95% CI	-1.58 ~ 1.77	−4.31 ~ .09	--6.02 ~ −1.10
Eta squared	0.00	0.00	0.01
Smoking status			
Current smoker	68.48 ± 7.32	64.76 ± 13.68	58.85 ± 18.18
Non-smoker	67.48 ± 10.20	65.92 ± 13.05	65.82 ± 14.16
t	0.814	−0.718	−3.880
*P* value	0.416	0.473	0.000
95% CI	-1.4 ~ 3.42	−4.34 ~ 2.02	−10.51 ~ −3.45
Eta squared	0.002	0.000	0.019
Alcohol consumption			
Drinker	68.12 ± 10.26	65.35 ± 12.83	63.89 ± 15.09
Non-drinker	67.32 ± 9.85	66.02 ± 13.23	65.75 ± 14.50
t	1.042	−0.662	−1.648
*P* value	0.298	.508	0.100
95% CI	-0.71 ~ 2.31	−2.65 ~ 1.31	−4.08 ~ .36
Eta squared	0.00	0.007	0.013
Financial status			
≤ 1000	68.86 ± 9.89	68.35 ± 13.75	66.95 ± 14.71
1000 ~ 2000	67.83 ± 10.31	65.7564 ± 13.15	64.8711 ± 15.03
> 2000	64.79 ± 18.52	62.1332 ± 11.04	63.5172 ± 13.42
F	7.578	9.795	2.566
*P* value	0.001	0.000	0.077
Eta squared	0.019	0.0204	0.006
Nationality			
Chinese	65.03 ± 8.01	66.25 ± 12.99	66.5289 ± 12.43
International	71.89 ± 11.41	65.07 ± 13.29	62.9035 ± 17.68
t	−9.10	1.234	3.105
*P* value	0.000	0.217	0.002
95% CI	-8.34 ~ −5.38	−0.70 ~ 3.07	1.33 ~ 5.92
Eta squared	0.094	0.002	0.012

### Socio-demographic characteristics and SRH of college students in Dalian City, China

#### Physiological health

The score of physiological subscale was significantly related to BMI, level of education, and financial status. Students with low BMI perceived their physiological health better than those with high BMI. Students in lower to moderate financial status perceived their health status better than those in high financial status (Table 3).

#### Psychological health

The score of psychological subscale was significantly related to BMI, and financial status (Table 3).

#### Social health

The score of social subscale was significantly related to type of institution, smoking status and level of education (Table 3).

#### Individual factors predicting health promoting lifestyle

Table 4 indicates that financial status negatively predicted health-promoting lifestyle among the Chinese students yielding low (adjusted R^2^ *=* 0.014) predictive explanatory power. In the international group, HPL was significantly associated with students’ major (adjusted R^2^ = 0.035), age (adjusted R^2^ =0.052) and level of education (adjusted R^2^ = 0.061).

**Table 4:** Stepwise multiple regression predictors of HPL among college students, at Dalian city, China

Group	Variable	B	SD Error	β	t	p	Tolerance	Variance inflation Factor
Chinese students	Financial status >2000 RMB	-0.128	0.045	−0.126	−2.838	0.005	0.984	1.016
International students	Age	0.028	0.007	0.240	4.021	0.000	0.893	1.120
	Student major	0.180	0.077	0.0132	2.339	0.020	0.995	1.005
	Level of education (post-graduate)	−0.209	0.105	−0.119	−1.988	0.048	0.889	1.125

#### Individual factors predicting self-rated health status

Table 5 shows the association between the individual factors and the three dimensions of SHMS V1.0.

**Table 5:** Stepwise multiple regression predictors of HPL among college students

Group	SHMV1.0 domain	Predictor	B	SD Error	β	t	p	Tolerance	VIF
Chinese	Physiological Health	BMI-24 ~ 27	−3.633	0.916	−0.174	−3.966	0.000	1.000	1.000
		Financial status ≤1000RMB	-1.92	0.704	−0.119	−2.73	0.007	0.997	1.000
	Psychological Health	Financial status ≥2000	−4.147	1.357	−0.138	−3.055	0.002	0.870	1.150
		BMI = 25-29.9	−4.363	1.484	−0.129	−2.940	0.003	0.917	1.091
		Financial status ≤1000	4.793	1.370	0.156	3.499	0.001	0.893	1.119
		Smoking status	9.267	3.462	0.113	2.677	0.008	0.992	1.009
		Gender	−2.795	1.150	−0.107	−2.430	0.015	0.914	1.094
	Social Health	Financial status ≤1000	4.592	1.297	0.156	3.540	0.000	0.994	1.006
		BMI = 25-29.9	−2.880	1.424	−0.089	−2.023	0.044	0.994	1.006
International students	Physiological health	BMI = 25-29.9	−4.687	2.360	−0.112	−1.986	0.048	0.996	1.004
		Age	0.566	.195	0.164	2.896	0.004	0.989	1.012
		Students’ major(medical)	5.368	2.274	0.133	2.360	0.019	0.997	1.003
		Smoking (smoker)	−3.574	1.629	−0.125	−2.194	0.029	0.988	1.012
	Psychological health	BMI = 25-29.9	−6.083	2.806	−0.125	−2.168	0.031	0.925	1.081
		Gender	4.115	1.636	0.145	2.516	0.012	0.926	1.080
		Age	0.984	0.223	0.245	4.413	0.000	0.997	1.003
		Students’ major(medical)	6.290	2.607	0.134	2.413	0.016	0.997	1.003
	Social health	Level of Education (Senior students)	−10.95	2.291	−0.262	−4.778	0.000	0.992	1.008
		Smoking (smoker)	−7.494	2.441	−0.168	−3.070	0.002	0.991	1.010
		Students’ Major (medical)	9.827	3.409	0.158	2.883	0.004	0.999	1.001

#### Physiological health

Physiological health among the Chinese students was negatively and positively associated with BMI (adjusted R^2^ =0.029) and financial status (adjusted R^2^ = 0.040). Besides, BMI (adjusted R^2^ = 0.025), age (adjusted R^2^ = 0.038) students’ major (adjusted R^2^ = 0.051), and smoking (adjusted R^2^ = 0.061) emerged as strong predictors of physiological health among the international students.

#### Psychological health

Financial status >2000 (adjusted R^2^ = 0.045), BMI = 25.0 - 29.9 (adjusted R^2^ =0.068), financial status ≤1000 (adjusted R^2^ = 0.089), smoking (adjusted R^2^ =0.100), and gender (adjusted R^2^0  = .109), predicted psychological health among the Chinese students. BMI = 25.0 - 29.9 (adjusted R^2^ = 0.056), gender adjusted (R^2^ = 0.069), age adjusted R^2^ =0.078), and students’ major (adjusted R^2^ =0.090), predicted psychological health among international students.

#### Societal health

Financial status ≤1000 (adjusted R^2^ =0.025) and BMI = 25-29.9 (adjusted R^2^ = 0.031) emerged as the predictors for social health among the Chinese students. Bachelors education (adjusted R^2^ = 0.074), smoking (adjusted R^2^ = 0.097) and students’ major (adjusted R^2^ = 0.119), emerged as strong predictors for social health in the international group.

#### Relationship between health-promoting behaviors and SRH status among college students in Dalian City, China

**Fig. 3:**
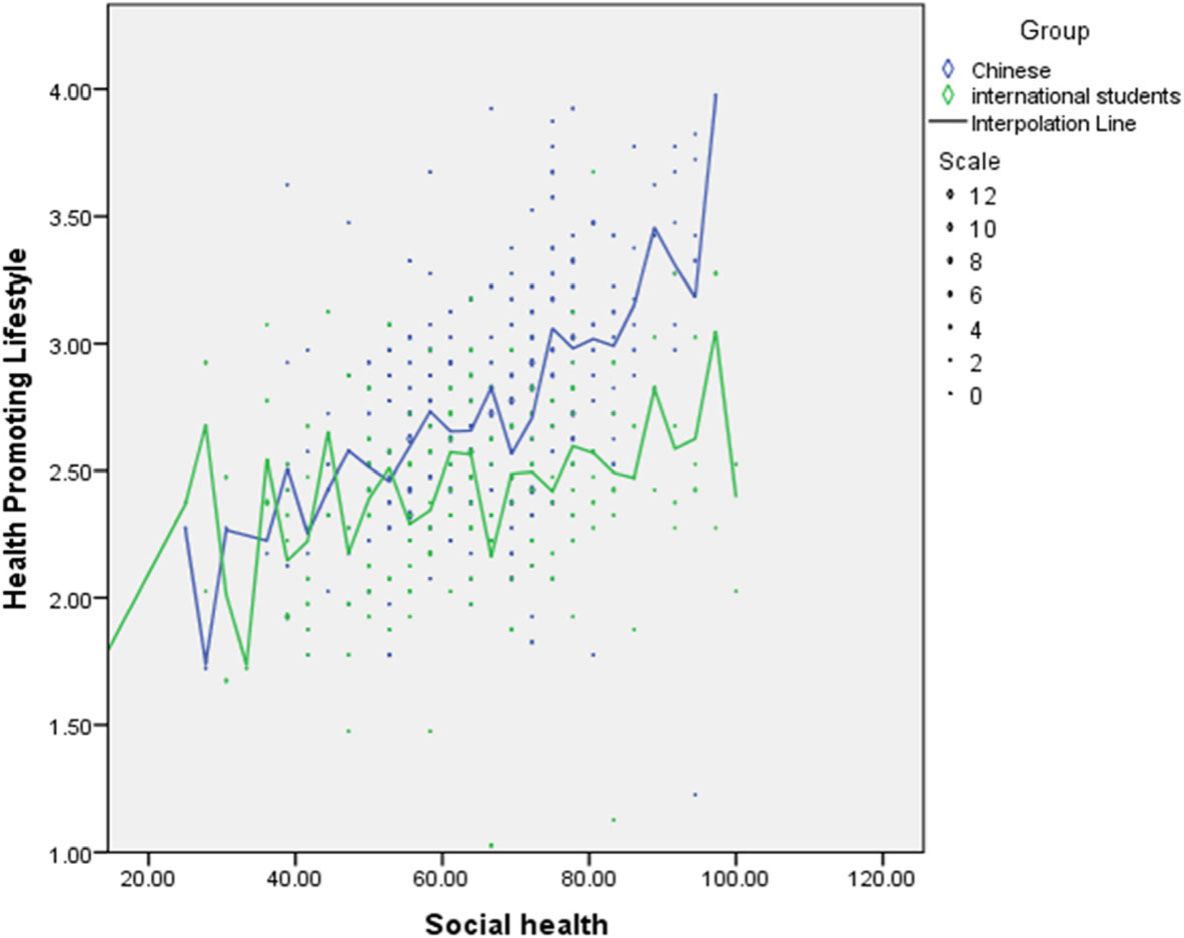
A scatterplot of social health and HPLP-II score in the Chinese and the international group

Total HPLP-II and all its subscales were correlated with the three SHMS V1.0 sub-scales among the Chinese college students. Physiological health (*r* = 0.34, *P* < 0.001), psychological health (*r* = 0.44, *P* < 0.001) and societal health (*r* = 0.48, *P* < 0.001) had a moderate to strong positive relationship with the average total HPLP-II score. In the international group, the average total HPLP-II correlated with the psychological health (*r* = 0.27, *P* < 0.001) and societal health (*r* = 0.296, *P* < 0.001). There was no significant association between the physiological health and the average total HPLP-II (*P* = 0.372) in the international group. Table 6 summarizes the correlation between HPLP-II score and SHMS V1.0 sub scales. Figure 3 shows a scatterplot of social health and HPLP-II score among the college students in Dalian City, China as an example that illustrates the strength of the positive relationships between the two variables (Table 6).

**Table 6:** Correlation between HPLP-II dimensions and SRH status among college students in Dalian City, China. (*n* = 829)

Variables	Physical Health		Psychological Health		Social Health	
	r	P	r	P	r	P
Chinese group (*N* = 504)						
Health R	0.193	0.000	0.222	0.000	0.246	0.000
Spiritual Growth	0.328	0.000	0.434	0.000	0.453	0.000
Physical Activity	0.246	0.000	0.296	0.000	0.274	0.000
Nutrition	0.240	0.000	0.320	0.000	0.342	0.000
Interpersonal R	0.307	0.000	0.433	0.000	0.537	0.000
Stress Management	0.300	0.000	0.387	0.000	0.409	0.000
Total HPLP-II Score	0.341	0.000	0.440	0.000	0.478	0.000
International group (*N* = 296)						
Health R	−0.161	0.006	0.063	0.000	0.067	0.253
Spiritual Growth	0.272	0.000	0.406	0.000	0.397	0.000
Physical Activity	−0.038	0.519	0.111	0.056	0.052	0.371
Nutrition	−0.081	0.167	0.162	0.050	0.147	0.012
Interpersonal Relations	0.198	0.010	0.339	0.000	0.440	0.000
Stress Management	0.047	0.424	0.162	0.000	0.218	0.000
Total HPLP-II Score	0.052	0.372	0.270	0.000	0.296	0.000

The logistic regression analysis was performed to assess the influence of HPL dimensions on SRH status which was a dichotomous outcome (Healthy and Sub-Optimal health status). The model contained six HPLP-II dimensions and adjusted for socio-demographic factors. The full model containing all the predictors was statistically significant X^2^ (17, *N* = 781) = 124.34, *P* < 0.001), indicating that the model was able to distinguish the respondents who were in healthy and Sub-Optimal Health Status. The model as a whole explained between 14.7% (Cox and Snell R square) and 20.4% (Nagelkerke R Square) of the variance in SRH status and correctly classified 69.3% of the cases. As shown in the Table 7, three of the HPLP-II dimensions made a statistically significant contribution to the model (physical activity, nutrition, and interpersonal relations). Based on overall lifestyle evaluations, compared with the participants with a healthy lifestyle, after demographic adjustment, subjects with a “moderate” lifestyle were at a 4.5 times at risk of developing SHS (OR: 4.5, 95% CI: 2.0–9.90) and those with a “good” lifestyle were at a 3.3 times higher risk of SHS (OR: 3.3, 95%CI: 1.500–7.18). Good and moderate HPLP-II levels of nutrition are associated with low risk of suboptimal health status (OR: 0.41 and 0.25, respectively). Participants in good and moderate HPLP-II levels of interpersonal relations are associated with higher risk of suboptimal health (OR:2.7 and 3.01, respectively) than those in excellent levels of HPLP-II (Table 7).

**Table 7:** Associations between health status and health-promoting lifestyle

Health promoting life profile	Healthy	SHS		95% Confidence Interval		
	N	N	OR*	Lower Limit	Upper Limit	P
Overall Evaluation						
Poor (13)	12	1	0.591	0.066	5.332	0.640
Moderate (296)	176	120	4.508	2.034	9.990	0.000
Good (456)	303	153	3.282	1.500	7.180	0.003
Excellent Ref^a^ (64)	56	8				0.000
Health Responsibility						
Poor (72)	38	34	6.55	0.683	62.884	0.100
Moderate (481)	320	161	3.89	0.432	35.066	0.220
Good (253)	167	86	5.29	0.597	46.866	0.130
Excellent Ref^a^(23)	22	1				0.080
Spiritual Growth						
Poor (14)	13	1	0.33	0.035	3.214	0.340
Moderate (160)	81	79	3.92	1.797	8.557	0.000
Good(531)	352	179	1.61	0.845	3.079	0.150
Excellent Ref^a^ (124)	101	23				0.000
Physical Activity						
Poor (46)	29	17	2.09	0.402	10.821	0.380
Moderate (451)	272	179	2.5	0.573	10.933	0.220
Good (302)	219	83	1.36	0.321	5.794	0.670
Excellent ref^a^ (30)	27	3				0.020
Nutrition						
Poor (16)	15	1	0.03	0.003	0.391	0.010
Moderate (295)	198	97	0.25	0.109	0.583	0.000
Good (445)	283	162	0.41	0.192	0.882	0.020
Excellent ref^a^ (73)	51	22				0.002
Interpersonal Relations						
Poor (8)	7	1	9.47	0.374	23.798	0.170
Moderate(185)	115	70	2.7	1.100	6.659	0.030
Good (543)	342	201	3.01	1.389	6.515	0.010
Excellent Ref^a^ (93)	82	11				0.040
Stress Management						
Poor (10)	9	1	0.51	0.580	3.364	0.64
Moderate(260)	162	98	1.4	0.623	2.988	0.46
Good (478)	310	168	1.36	0.931	1.075	0.44
Excellent Ref ^a^(81)	66	15				0.764

^a^Demographic variables adjusted for included age, gender, BMI, education level, drinking, Smoking, Financial Status

Ref^a^ is the reference group

## Discussion

The HPLP-II scores indicated that both the Chinese and the international students practiced healthy behaviors in this study. However, there was a significant difference in the overall HPLP-II mean score and in all the subscales between the two groups. Chinese students practice healthy behaviors more than international students. This could be attributed to the fact that Chinese culture favors Chinese students in all aspects of health-promoting lifestyle. Culture influences the value placed on health and health-promoting lifestyles [17]. Furthermore, Chinese students are not only aware of socio-cultural activities but also access support and motivation from family/friends that might encourage them to pay more attention to adopting a healthy lifestyle [27]. On the contrary, the international students’ unfamiliarity with the environmental/social differences and the health care services in China might negatively affect their participation in an HPL.

Notably, our findings also indicated that the two groups differed in the least adopted health-promoting behaviors. The lowest subscale in the Chinese group was health responsibility. This finding is consistent with those of previous studies in China [7, 28]. The lowest subscale in the international group was observed in the stress management. Our finding is consistent with those of previous studies [29]. International students process and manage their daily requirements through financial expenses (e.g. ordering food and beverages through phone calls, hiring private taxi drivers for shopping and moving around the school etc.), which eventually lead to unworthy expenses, poor lifestyle, and private life. Stress management involves one’s ability to change a stressful situation, deal with problems, take care of themselves, and make time for rest and relaxation. Previous research has proposed that it is not the presence of stressors but rather inability to cope effectively with stressful situations that have unfavorable consequences for health status [30]. Therefore, the universities should encourage international students and Chinese to participate in common extracurricular activities within the university to empower their social life in order to cope effectively with stressful situations.

Individual factors have been shown to influence health promotion and lifestyle [31–33]. This current study found that international post-graduate medical students engaged less frequently in HPL. Our findings contradict the findings reported by Wang et al. [34]. A medical degree is often regarded as a particularly demanding program. Most medical students almost entirely spend time to study with very little time left for relaxation and co-curriculum activities. Relaxation techniques have proven to be particularly an effective stress management intervention in (medical) student populations [35].

Risk behaviors, particularly smoking was found to be higher in international students and was negatively associated with their physiological and social health. This could be attributed to poor stress management strategies and less interaction with their environment. Social deinstitutionalization can keep international students constantly to interact with their devices (smartphones or computers) which may consequently lead to less physical activity.

Though the Chinese student’s group had lower BMI than international students, high BMI score and high financial status were negatively associated with physiological and psychological health status. Impaired body image concerns a person’s perception, feelings, and thoughts about his or her body can prevent the exposure and participation in the health promoting activities, particularly physical activity. Poor self-image and a low self-esteem can lead to low degree of satisfaction about oneself in terms of size, shape and general appearance among overweight/obese students [36]. Thus, the psychological instability that associates with body shape and size may contribute to the physical inactivity, social withdrawal, and sub optimal health status. Overweight/obesity may lead to some risk factors of cardiovascular and metabolic derangements such as an increase in elevated blood pressure and blood lipid profiles. Also, the literature shows that the students with higher income levels had increased dietary intake; which may promote the risk of gaining weight and high risk of suboptimal health. The Chinese students who had lower BMI and financial status perceived health better than other Chinese students in this study. A university-based health education program aimed at promoting healthy lifestyles and engaging students in regular medical check-ups are needed to prevent cardio-metabolic risks among the Chinese students.

This study found that good and moderate HPLP-II levels of interpersonal relations were associated with an increased risk of SHS. On the other hand, good and moderate HPLP-II levels of nutrition were associated with lower risk of suboptimal health status in the present study. This study merits further study to consider clinical and laboratory results such as blood pressure, electrocardiography (ECG), lipid profiles and glucose levels to identify the cardio-metabolic risks among the college students.

To the best of our knowledge, this is the first study reporting on the health-promoting lifestyle and self-rated health among college students in terms of differences between international and Chinese students. Therefore, it provides vital information related to health-promoting lifestyle to universities in China, to better support various students.

Certain limitations of the study should be taken into account, especially its cross-sectional design, which limits any causal conclusions. In addition, the data were self-reported and questionnaires were available in only two languages (Chinese and English). To fully accommodate all international students, it would be of great interest to provide other language options. Data were based on self-reports, which are sensitive to social desirability biases meaning that social pressures might interfere with these results.

## Conclusion

Collectively, these findings provide a convincing body of evidence to support the role of socio-cultural factors that affect the HPL and SRH of college students. Policy makers should be aware of socio-cultural influences when designing programs to promote health among students from different ethnic/group. Importantly, to reduce chronic illnesses more effectively and improve population health, health education programs should be planned to stimulate the interests of different students according to their socio-cultural characteristics.

## Abbreviations

ANOVA: Analysis of variance
BMI: Body mass index
HPL: Health-promoting lifestyle
HPLP-II: Health-promoting lifestyle profile II
SHS: Suboptimal health status
SHMS V1.0: Suboptimal health measurement scale version 1.0
SRH: Self-rated health status
